# Bioactive Peptides from Muscle Sources: Meat and Fish

**DOI:** 10.3390/nu3090765

**Published:** 2011-08-31

**Authors:** Joseph Thomas Ryan, Reynolds Paul Ross, Declan Bolton, Gerald F. Fitzgerald, Catherine Stanton

**Affiliations:** 1 Teagasc Food Research Centre, Moorepark, Fermoy, Co. Cork, Ireland; Email: josephthomas.ryan@teagasc.ie (J.T.R.); paul.ross@teagasc.ie (R.P.R.); 2 Alimentary Pharmabiotic Centre, Biosciences Institute, University College Cork, Co. Cork, Ireland; Email: g.fitzgerald@ucc.ie; 3 Teagasc Food Research Centre, Ashtown, Co. Dublin, Ireland; Email: declan.bolton@teagasc.ie; 4 Department of Microbiology, University College Cork, Co. Cork, Ireland

**Keywords:** angiotensin converting enzyme inhibitors, bioactive peptides, meat, fish

## Abstract

Bioactive peptides have been identified in a range of foods, including plant, milk and muscle, e.g., beef, chicken, pork and fish muscle proteins. Bioactive peptides from food proteins offer major potential for incorporation into functional foods and nutraceuticals. The aim of this paper is to present an outline of the bioactive peptides identified in the muscle protein of meat to date, with a focus on muscle protein from domestic animals and fish. The majority of research on bioactives from meat sources has focused on angiotensin-1-converting enzyme (ACE) inhibitory and antioxidant peptides.

## 1. Introduction

Food proteins have long been recognized for their nutritional and functional properties. The nutritional properties of proteins are associated with their amino acid content in conjunction with the physiological utilization of specific amino acids upon digestion and absorption [1,2]. On the other hand, the functional properties of proteins relate to their contribution to the physiochemical and sensory properties of foods [2,3]. In recent years, a considerable amount of research has also focused on the liberation of bioactive peptides which are encrypted within food proteins, with a view to utilizing such peptides as functional food ingredients aimed at health maintenance. 

Bioactive peptides have been defined as “food derived components (genuine or generated) that, in addition to their nutritional value exert a physiological effect in the body” [4]. Interestingly, within the parent protein sequence, the peptides are inactive and thus must be released to exert an effect. These bioactive peptides are usually 2–20 amino acid residues in length, although, some have been reported to be >20 amino acid residues. Bioactive peptides may be absorbed through the intestine where they subsequently enter the circulatory system intact to exert various physiological effects, or they may produce local effects in the digestive tract [5]. Food derived bioactive peptides have been shown to display a wide range of physiological functions including antihypertensive, antioxidative, opioid agonistic, immunomodulatory, antimicrobial, prebiotic, mineral binding, antithrombotic and hypocholesterolemic effects [6]. 

Meat and fish provide valuable sources of protein for many populations around the world; furthermore, meat and fish proteins offer huge potential as novel sources of bioactive peptides. To date, bioactive peptides displaying antihypertensive, antioxidant, antimicrobial and antiproliferative effects have been found in the hydrolysates of meat and fish proteins [7,8,9,10].

### History of Bioactive Peptide Discovery

The first food-derived bioactive peptide was identified in 1950 when Mellander reported that casein phosphorylated peptides enhanced vitamin D-independent bone calcification in rachitic infants [11]. However, interest in this field has increased considerably in the last two decades, with the majority of research focusing on the identification of bioactive peptides from milk proteins [12,13]. Bioactive peptides are inactive or latent in the parent protein but are released in an active form after proteolytic digestion [14]. Methods employed in the proteolytic digestion of parent proteins include hydrolysis by digestive enzymes [15], plant and bacterial proteases [16,17], and following microbial fermentation [18,19,20].

The most widely reported bioactive peptides from milk and other food sources display antihypertensive activity, particularly those peptides which inhibit the action of angiotensin-1-converting enzyme (ACE) [21,22]. Indeed, currently a number of commercial products which contain such peptides have been released onto the market. Perhaps the two most widely known products available are both fermented milk-based products, *i.e.*, Calpis^®^ and Evolus^®^. Calpis^®^ is a sour milk product from Japan which contains the antihypertensive peptides VPP and IPP both derived from milk caseins. Evolus^®^ is a Finnish product that claims to reduce blood pressure. Evolus^®^ also contains the peptides VPP and IPP which correspond to the fragments of β-casein f (84–86) and κ-casein f (74–76) [14]. Various milk protein hydrolysates which claim to contain bioactive peptides are also available as food ingredients. For example C12^®^, a casein derived peptide supplied by DMV International, claims to reduce blood pressure. 

## 2. Production of Bioactive Peptides

While numerous methods have been utilized to release bioactive peptides from food proteins, enzymatic hydrolysis of whole protein is the most widely used technique. For example, a number of bioactive peptides have been isolated from meat using digestive enzymes such as pepsin, trypsin and chymotrypsin. Indeed, peptic digestion of porcine skeletal muscles has resulted in the generation of numerous ACE inhibitory peptides [23,24]. Various proteases from bacterial, animal and plant origin have also been used to generate bioactive peptides from meat sources [10,25,26].

Several researchers have described the isolation of bioactive peptides following bacterial fermentation of milk proteins where lactobacilli have been commonly used [27,28,29]. However, microbial fermentation of meat proteins has been less successful, presumably due to the poor proteolytic activity of the lactobacilli used in meat fermentations [30,31]. Indeed, to the best of our knowledge, no bioactive peptides have been produced from the microbial fermentation of muscle proteins.

### Identification of Bioactive Peptides

**Figure 1:**
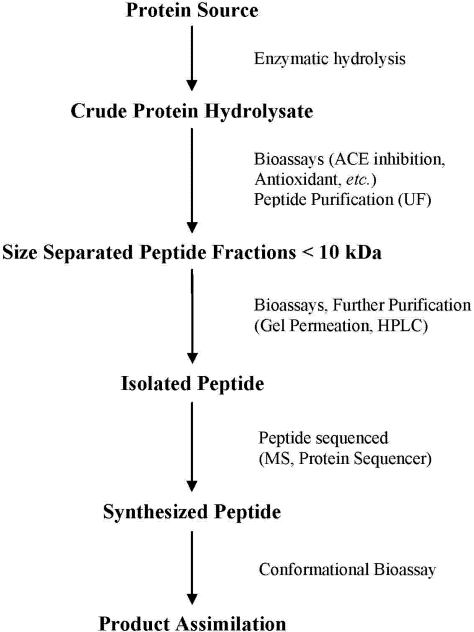
Procedure for the isolation and identification of bioactive peptides from food proteins, adapted from Arihara & Ohata [30].

Following the hydrolysis of the protein substrate (Figure 1), the hydrolysates are assayed for various bioactivities. After the detection of bioactivity within the crude protein hydrolysates, the hydrolysates are then fractionated based on peptide size, which is typically performed using ultrafiltration [32,33,34]. The hydrolysate fraction displaying the highest bioactivity is then further purified to separate individual peptides using different techniques, most notably reverse phase high performance liquid chromatography (RP-HPLC) or gel permeation chromatography [15,22,35]. Individual peptide fractions are identified using the combined techniques of mass spectrometry and protein sequencing. Lastly, a synthetic version of the peptide is synthesized and the assay is repeated to verify bioactivity [21,36,37].

## 3. Antihypertensive Peptides

Bioactive peptides have been found in a wide range of foods, and as already mentioned, the most extensively researched of these are the antihypertensive peptides, particularly those which inhibit ACE [3,22]. ACE inhibitory peptides were first discovered in snake venom [38] and since then numerous synthetic ACE inhibitors have been produced, with Captopril being the most common. Additionally, Captopril and other synthetic ACE inhibitors are known to exert various side-effects such as coughing, taste disturbances and skin rashes [35,39]. These side effects, coupled with the fact that hypertension affects one third of the Western worlds’ population and is a known risk factor for stroke and cardiovascular disease, has contributed to the ongoing search for food derived antihypertensive peptides for exploitation as antihypertensive agents in functional foods and nutraceuticals [40,41].

### 3.1. Mechanism of ACE Inhibition

ACE, or kininase ІІ, is a dipeptidyl carboxy peptidase (EC 3.4.15.1) found in various tissues in the body and is integral to the moderation of blood pressure and normal heart function [40]. In the rennin-angiotensin system, ACE catalyses the conversion of the inactive form of angiotensin I (Ang І) to the potent vasoconstrictor angiotensin II (Ang ІІ). Additionally, ACE is involved in the deactivation of the hypotensive peptide, bradykinin [32]. This is as a result of ACE cleaving the *C*-terminal dipeptide from Ang І (His-Leu) and bradykinin. Ang ІІ is a documented potent vasoconstrictor which acts directly on vascular smooth muscle cells. Ang ІІ is also responsible for the expansion of vascular volume via sodium retention and fluid retention [42,43,44,45]. Bradykinin is responsible for uterine and ileal smooth muscle contraction, enhanced vascular permeability, activation of peripheral and C fibers and increases in mucous secretion [42,46]. Furthermore, and more notably bradykinin contributes to vasodilation by advancing the assembly of arachidonic acid metabolites, nitric oxide, and endothelium-derived hyperpolarizing factor in the vascular endothelium [42,47]. Therefore, ACE inhibitors function by maintaining the balance between the associated vasoconstrictive and salt-retentive attributes of Ang ІІ with the vasodilatory effect of bradykinin. The balance is maintained by decreasing the production of Ang ІІ and reducing the degradation of bradykinin [42]. While synthetic ACE inhibitors, such as Captopril function by directly blocking the action of ACE, ACE inhibitory peptides function by reacting with ACE, thus ACE is unavailable to cleave Ang І and prevent the production of the vasoconstrictor Ang ІІ [48]. In the past decade, it has been reported that muscle proteins from animals are a viable source of ACE inhibitory peptides *in vitro* and may potentially be incorporated into nutraceutical products to exert antihypertensive effects *in vivo* [10,25,49].

#### 3.1.1. ACE Inhibitor Type Peptides

An important factor when discussing ACE inhibitory peptides isolated from food proteins is the discrepancy between ACE inhibitory activity of peptides *in vitro* and their antihypertensive effect *in vivo* [3,30]. ACE inhibitory food peptides can be divided into three categories, depending on their inhibitory activity following preincubation with ACE [33]. The first group of ACE inhibitory peptides is known as “true inhibitor type” peptides. The IC_50_ value of these peptides is not affected by preincubation with ACE. The second category of peptides, known as “substrate type” peptides are hydrolyzed by ACE resulting in weak inhibitory activity. Finally, the third category is “pro-drug type” inhibitors. The peptides in this category are converted to “true inhibitor type” peptides by ACE or proteases of the digestive tract. *In vivo* studies have demonstrated that only peptides belonging to the groups of true inhibitor type or pro-drug type reduce the systolic blood pressure of spontaneously hypertensive rats (SHR) [30]. 

To date, the majority of ACE inhibitory peptides found in meat can be classified as true inhibitor type peptides [10,50,51]. These peptides may act in one of two ways, first the peptide may bind to the active site of the ACE enzyme or second it may bind to an inhibitor site located on the ACE enzyme thereby modifying the protein confirmation and preventing the substrate (Ang І) from binding to the enzyme active site [52].

#### 3.1.2. Absorption of ACE Inhibitory Peptides

When determining or extrapolating the *in vivo* efficacy of potential ACE inhibitory peptides in humans using the SHR model, differences in bioavailability of nutrients between both species is an important consideration. Bioavailability is the term used to “express the proportion of the total amount of a nutrient that can be absorbed and utilized” [53]. The bioavailability of a nutrient is in part controlled by the physicochemical properties of the nutrient; these include molecular weight and size, lipophilicity, p *K*_a_, charge and solubility. Factors which affect the variability of nutrient absorption and which are species-dependent include the physiological factors of pH, gastrointestinal (GI) motility, transit time, intestinal permeability as well as the diverse groups of enzymes and transporters [54,55,56]. It has been proposed that the larger relative absorptive surface area of the human GI tract results in faster absorption of nutrients and a larger uptake than that of the rat [57]. Finally, when extrapolating the results from *in vivo* studies of SHR to human subjects, it is important to incorporate dosage of nutrient, form of nutrient, period of administration, metabolic state of subjects and control diets used [58].

For an ACE inhibitory peptide to function *in vivo*, it must first enter the circulatory system intact. Peptides may be hydrolyzed by enzymes of the digestive tract as well as peptidases associated with the brush border membrane, cytoplasm or serum [59,60]. To date, studies documenting the intestinal absorption of ACE inhibitory peptides *in vitro* have employed the Caco-2 cell monolayer [61], as it is generally acknowledged that this cell line is a suitable model to predict the permeability of intestinal epithelial cells to pharmaceuticals [62,63,64]. Interestingly, the occurrence of peptides which demonstrate ACE inhibitory activity *in vitro* but poor antihypertensive activity *in vivo* may be explained by the possible alteration of peptides prior to reaching the target ACE enzyme in the circulatory system [62,65]. The opposite has also proven true, in that peptides determined to have poor ACE inhibitory activity *in vitro* have demonstrated a significant systolic blood pressure (SBP) lowering effect *in vivo*, possibly due to intestinal modification [23,66].

The bioavailability of the β-casein ACE inhibitory peptide LHLPLP was determined by evaluating it’s resistance to brush border peptidases, serum peptidases as well as identifying the mechanism of transepithelial transport [67]. It was concluded that LHLPLP was partly hydrolyzed to HLPLP by brush border peptidases and HLPLP was rapidly absorbed by the Caco-2 cell monolayer in a concentration-dependent manner. Furthermore, the peptide remained “practically intact” after one hour incubation with human plasma and remained unbound to plasma proteins. This suggests that the peptide LHLPLP is hydrolyzed to HLPLP, the minimum active form, which is then readily absorbed across the intestinal epithelium by paracellular diffusion and is stable to serum proteases. This offers the possibility that HLPLP may be a viable alternative in the treatment or prevention of hypertension in humans.

The well documented ACE inhibitory milk peptides IPP and VPP have also been subject to *in vitro* permeability studies [59]. Both peptides were found to be readily transported across the intestinal epithelium of the various models used. It has been reported that IPP avoids gastrointestinal digestion and can enter the circulatory system of humans intact [68]. Studies involving the oral administration of foods rich in both IPP and VPP have demonstrated that such foods exhibit blood pressure lowering effects on hypertensive patients [69,70,71]. This clearly demonstrates that certain ACE inhibitory peptides are sufficiently well absorbed by the body to be effective at reducing blood pressure of hypertensive subjects. The challenge for researchers now is to identify bioactive peptides *in vitro* which can enter the circulatory system intact and remain active at their target site.

#### 3.1.3. Structure Correlation of ACE Inhibitory Peptides

As stated previously, the mode of action of the majority of ACE inhibitory peptides is thought to be as competitive substrates for ACE. The structure activity relationships of ACE inhibitory peptides has not yet been confirmed due to the assorted amino acid sequence properties of ACE inhibitory peptides identified to date [21,30]. The tripeptide sequence at the *C*-terminal end of ACE inhibitory peptides has been suggested as the controlling factor of ACE inhibitory peptides, which regularly contains hydrophobic amino acid residues. It has been reported that at the penultimate positions, aliphatic (V, I, and A), basic (R) and aromatic (Y and F) residues are preferred, while aromatic (W, Y, and F), proline (P) and aliphatic (I, A, L and M) residues are favored at the ultimate position of the *C*-terminal end of the peptide [33,72,73,74,75,76]. This is because of the interaction of these residues with the three hydrophobic sub-sites located on the active site of ACE [49,77]. The positive charge associated with arginine at the *C*-terminus has also been associated with the ACE inhibitory activity of some peptides, as has the presence of lysine (K) which possesses a positive charge on the є-amino group at the *C*-terminus [27]. Another common feature of ACE inhibitory peptides is the hydrophobic nature of the *N*-terminal end of the peptide [52,78]. In the case of peptides greater than three amino acids in length, it has been suggested that the presence of hydroxyproline (Hyp) is critical for the binding of peptides to the active sites of ACE [79]. Furthermore, the overall hydrophobicity of the peptide is important. Hydrophilic peptides possess weak or no ACE inhibitory activity, since hydrophilic peptides are incompatible with the active sites of ACE. Indeed, inhibition of ACE is achieved by hydrophobic peptides which display high affinity to the active sub-sites of ACE [8,21,80].

### 3.2. Antihypertensive Peptides from Animal Sources

The generation of force (*i.e.*, contraction) by skeletal muscle is the responsibility of the muscle proteins, actin and myosin. Both of these proteins are associated with two distinct types of muscle filaments. Myosin proteins are associated with the thick filaments of skeletal muscle while actin is associated with the thin filaments of skeletal muscle. Also present in the thin filaments are the proteins troponin and tropomyosin [3,81]. In striated muscle, the myosin thick filaments extend along the thin filaments of actin and are known as sacromeres. Sacromeres are further longitudinally repeated to form myofibrils. Myofibrils are then arranged in parallel to form myofibers, these myofibers then form muscle fibers. The muscle proteins arranged in parallel bands result in skeletal and cardiac muscle being striped or striated in appearance [3,81]. Contraction of striated muscle is due to the interaction of the thick and thin filaments. Nerve impulses release Ca^2+^ from the sacroplasmic reticulum, which pass through the myofibrils resulting in the sliding of myosin and actin filaments over one another, causing the sacromeres to shorten resulting in muscle contraction [82]. Interestingly, ACE inhibitory peptides have been identified in the hydrolysates of actin, myosin and troponin [50,83,84].

#### 3.2.1. Antihypertensive Peptides from Myosin Sources

Arihara and colleagues [23] identified two ACE inhibitory pentapeptides from the thermolysin digestion of porcine myosin. These were named myopentapeptides A and B and their sequences determined. The amino acid sequence of myopentapeptide A was found to be MNPPK, which corresponded to positions 79–83 on the myosin heavy chain, while myopentapeptide B, with the amino acid sequence ITTNP, corresponded to positions 306–310 on the myosin heavy chain. The antihypertensive activities of myopentapeptide A and myopentapeptide B were investigated in SHR, in addition to other thermolysin hydrolysates of porcine skeletal muscle proteins. Administration of myopentapeptide A and myopentapeptide B at a concentration of 1 mg per kilogram of animal weight resulted in a maximum decrease in SBP of 23.4 ± 3.0 mmHg and 21.0 ± 3.1 mmHg after 6 h, respectively. At 24 h, the SBP of both test groups was still significantly lower than that of the control group, indicating that both myopentapeptide A and myopentapeptide B are potent antihypertensive peptides *in vivo* [66]. 

Another ACE inhibitory octapeptide VKKVLGNP was discovered corresponding to positions 47–54 on the myosin light chain [84]. This peptide was generated following the digestion of crude myosin light chain with pepsin. The IC_50_ value of this peptide was calculated to be 28.5 µM. Following the administration of this purified peptide to SHR at a concentration of 10 mg per kilogram of animal weight, the SBP decreased up to 3 h post-administration with SBP returning to the pre administration value after 9 h. It was therefore postulated that the peptide VKKVLGNP was an effective hypotensor *in vivo*.

Porcine skeletal muscle also was found to be a source of antihypertensive peptides when crude myosin B was hydrolyzed with pepsin. [83]. Indeed, within this hydrolysate a novel peptide M6 was identified with the amino acid sequence KRVITY. This peptide corresponded to positions 191–196 on the myosin heavy chain. The SBP of SHR was immediately reduced following oral administration of M6 peptide with a maximum decrease of 23 mmHg after 6 h, with the SBP of test animals returning to the control after 9 h. This reduction in SBP of SHR would indicate that the M6 peptide is a potent hypotensor *in vivo*. Furthermore, the M6 peptide retained its ACE inhibitory activity after heating the myosin B to 98 °C for 10 min prior to hydrolysis by pepsin. Retention of ACE inhibitory activity after such thermal treatment clearly indicates that bioactivity is retained even after the cooking process.

#### 3.2.2. Antihypertensive Peptides from Troponin Sources

In a study to identify the most active ACE inhibitory peptides from porcine skeletal muscle, a bioactive peptide of 9 amino acids was released from the regulatory protein troponin C [85]. This peptide with the amino acid sequence RMLGQTPTK had an IC_50_ value of 34 µM and was categorized as being a non-competitive inhibitor [50]. Subsequently, the same researchers described a novel ACE inhibitory peptide from the pepsin hydrolysate of crude porcine troponin [24]. This peptide with the amino acid sequence KRQKYDI (corresponding to amino acid positions 198–204 of troponin T) had an IC_50_ value of 26.2 µM. Incubation of the peptide with ACE resulted in a decrease in the observed IC_50_ value suggesting that the peptide KRQHYDI is a substrate type inhibitor. Administration of the peptide to SHR resulted in temporary reduction of SBP. It has therefore been suggested that the peptide KRQHYDI may inhibit ACE activity *in vivo* in the short term.

As demonstrated in the above studies, bioactive peptides can be released from the digestion of meat proteins by various digestive enzymes. ACE inhibitory peptides were released following *in vitro* gastric simulated digestion of pork meat using a combination of the digestive enzymes pepsin and pancreatin [86]. The active ACE inhibitory peptides generated in this study were found to be homologous to the amino acid sequence of the muscle protein titin, representing the first documented ACE inhibitory peptides released from the myofibrillar protein titin. The amino acid sequence of the most active peptide present in this hydrolysate was identified as KAPVA, the IC_50_ value of this peptide was determined to be 46.56 µM.

#### 3.2.3. Antihypertensive Peptides from Collagen

Connective tissue, which functions to convert force (*i.e.*, contraction) into movement by attaching muscle to bone via robust strips of connective tissue called tendons, is mainly composed of fibrillar collagen. There are numerous collagens present in the extracellular matrix and they are the major structural constituent of connective tissue. All collagens contain repetition of the proline rich tripeptide Gly-X-Y; that forms the trimeric collagen triple helices [87].

Recently, it was reported that ACE inhibitory peptides were purified from the hydrolysate of bovine skin gelatine [34]. Five proteases were employed, Alcalase, α chymotrypsin, Neutrase, Pronase E, and trypsin. Hydrolysis was performed using sequential protease treatments and a three-step ultrafiltration membrane reactor. This involved collagen first being hydrolyzed by Alcalase, fractioned with a 10 kDa membrane, followed by hydrolysis with Pronase E, fractionated with a 5 kDa membrane and finally hydrolyzed with collagenase and separated using a 1 kDa molecular weight cut-off (MWCO) membrane. Two peptides responsible for ACE inhibitory activity were subsequently isolated from this low molecular weight fraction. The first peptide referred to as EIIICIII, with the amino acid sequence GPV had an IC_50_ value of 4.7 µM, while the second peptide known as EIIICIV, with amino acid sequence GPL had an IC_50_ value of 2.55 µM. 

Chicken breast muscle was identified as a potential source of ACE inhibitory peptides by Saiga *et al.* 2003 [79]. The researchers fed two chicken breast muscle extracts to SHR, the first an untreated chicken muscle extract, the second a chicken muscle extract hydrolyzed with an *Aspergillus* protease. The blood pressure of SHR was significantly lowered after ingestion of both extracts. A reduction in blood pressure occurred after 1 h and lasted until 4 h post-ingestion. The purification of ACE inhibitory peptides from *Aspergillus* protease- and gastric protease-hydrolyzed chicken breast muscle resulted in the identification of four peptides displaying strong ACE inhibitory effects. The peptide expressing the strongest ACE inhibitory activity referred to as P4 had amino acid sequence GFHypGTHypGLHypGF. The IC_50_ value of this peptide was determined as 42.4 µM, whose sequence along with the others identified was homologous to that of collagen [79]. Interestingly, an immediate decrease in the blood pressure of SHR was observed after intravenous administration of the P4 peptide (30 mg per kilogram of body weight). However, blood pressure returned to base pressure 60 min post administration, suggesting that the P4 peptide may not act as a long-term vasodepressor *in vivo* [36]. It was also demonstrated that the P4 peptide is a non-competitive inhibitor of ACE. The presence of an aromatic amino acid at the antepenultimate position and phenylalanine at the *C*-terminal end of the peptide are responsible for the ACE inhibitory effect of the peptide. This supports the theory that the ACE inhibitory activity of peptides is based on the presence of hydrophobic amino acids located at the *C*-terminal end of the peptide. 

ACE inhibitory peptides were also detected following the hydrolysis of chicken collagen by an *Aspergillus oryzae* protease. The resulting hydrolysate was further hydrolyzed with four proteases, namely protease FP, protease A, amino G and protease N. Four peptides displaying ACE inhibitory activity were identified along with their individual IC_50_ values, GAHypGLHypGP (IC_50_ 29.4 µM), GAHypGPAGPGGIHypGERG (IC_50_ 45.6 µM), GLHypGSRGERGERGLHypG (IC_50_ 60.8 µM) and finally GIHypGERGPVGPSG (IC_50_ 43.4 µM). The chicken collagen hydrolysates were fed to SHR and a reduction in blood pressure was detected 2 h after administration of the hydrolysates with the greatest reduction occurring after 6 h. Following continuous long-term administration of chicken collagen hydrolysates to SHR a significant reduction in blood pressure was observed. It is therefore proposed that low molecular weight chicken collagen hydrolysates show long-term hypotensive effects *in vivo* and have potential as antihypertensive therapeutic agents.

#### 3.2.4. Antihypertensive Peptides from Miscellaneous Sources

The first identified ACE inhibitory peptide from beef hydrolysate was reported to be a hexapeptide with the amino acid sequence VLAQYK; this peptide had an IC_50_ value of 32.06 µM and was isolated from the sacroplasmic proteins of beef rump [25]. Feeding this hexapeptide to SHR resulted in a significant reduction in SBP in addition to lower total and LDL cholesterol blood concentrations [88]. It was proposed that the hexapeptide VLAQYK is a potent ACE inhibitor with potential use in clinical applications and functional food products. Subsequently, the same research group explored the possibility that beef sacroplasmic protein hydrolysates may contain other peptides capable of ACE inhibitory activity. This was part of a larger investigation to determine if antihypertensive peptides were capable of exhibiting other bioactivities, namely antimicrobial and antiproliferative [10]. Four peptides were found to exhibit strong ACE inhibitory activity, the strongest of which was from the Alcalase hydrolyzed hydrolysate. Other bioactivities associated with these peptides will be discussed later. A summary of ACE inhibitory meat derived peptides is given in Table 1.

**Table 1:** ACE inhibitory peptides from meat of domestic animals. Adapted with permission from American Chemical Society [3], Copyright 2005.

Source	Amino Acid Sequence	Parent Protein	Enzyme	IC_50_ (µM)	SHR ^a^	Ref.
pig	ITTNP	myosin	thermolysin	549	∆ 21.0 mmHg	[23,66]
	MNPPK	myosin	synthesized	945.5	∆ 23.4 mmHg	[23,66]
	MNP	myosin	synthesized	66.6	∆ 19.6 mmHg	[23,66]
	PPL	myosin	synthesized	>1000	∆ 24.7 mmHg	[23,66]
	NPP	myosin	synthesized	290.5	∆ 17.6 mmHg	[23,66]
	TNP	myosin	synthesized	207.4	∆ 11.1 mmHg	[23,66]
	RMLGQTPTK	troponin	pepsin	34	nt	[50,85]
	RMLGQTP	troponin	pepsin	503	nt	[50]
	VKKVLGNP	myosin	pepsin	28.5	∆ 24.0 mmHg ^b^	[84]
	KRVITY	myosin	pepsin	6.1	∆ 23.0 mmHg ^b^	[83]
	VKAGF	actin	pepsin	20.3	∆ 17.0 mmHg ^b^	[83]
	KRQKYDI	troponin T	pepsin	26.2	∆ 9.9 mmHg ^b^	[24]
	KAPVA	titin	pepsin + pancreatin	46.56	nt	[86]
	PTPVP	titin	pepsin + pancreatin	256.41	nt	[86]
	ER	muscle	pepsin + pancreatin	667	nt	[86]
	KLP	muscle	pepsin + pancreatin	500	nt	[86]
	RPR	muscle	pepsin + pancreatin	382	nt	[86]
chicken	LKA	creatine kinase	thermolysin	8.5	nt	[89]
	LKP	aldolase	thermolysin	0.32	∆ 75 mmHg ^c^	[89]
	LAP	muscle	thermolysin	3.2	∆ 40.0 mmHg ^c^	[89]
	FQKPKR	myosin	thermolysin	14	nt	[89]
	FKGRYYP	creatine kinase	thermolysin	0.55	∆ 0 mmHg ^c^	[89]
	IKW	muscle	thermolysin	0.21	∆ 50.0 mmHg ^c^	[89]
	GFXGTXGLXGF	muscle	*Aspergillus* protease + gastric proteases	42.4	∆ 20 mmHg ^d^	[36,79]
	GAXGLXGP	collagen	*Aspergillus* protease + protease FP, A, G, N	29.4	nt	[37]
	GAXGPAGPGGIXGERGLXG	collagen	*Aspergillus* protease + protease FP, A, G, N	45.6	nt	[37]
	GLXGSRGERGERGLXG	collagen	*Aspergillus* protease + protease FP, A, G, N	60.8	nt	[37]
	GIXGSRGERGPVGPSG	collagen	*Aspergillus* protease + protease FP, A, G, N	43.4	nt	[37]
cow	VLAQYK	muscle	thermolysin + proteinase A	32.06	+	[25,88]
	GFHI	muscle	proteinase K	64.3	nt	[10]
	DFHING	muscle	alcalase	50.5	nt	[10]
	FHG	muscle	thermolysin + proteinase A	52.9	nt	[10]
	GLSDGEWQ	muscle	thermolysin + proteinase A	117	nt	[10]
	GPV	skin gelatin	alcalase + pronase E + collagenase	4.67	nt	[34]
	GPL	skin gelatin	alcalase + pronase E + collagenase	2.55	nt	[34]

^a^ The maximum decrease in systolic blood pressure in SHR upon oral administration of peptide at 1 mg/kg; ^b^ The maximum decrease in systolic blood pressure in SHR upon oral administration of peptide at 10 mg/kg; ^c^ The maximum decrease in systolic blood pressure in SHR upon intravenous administration of peptide at 10 mg/kg; ^d^ The maximum decrease in systolic blood pressure in SHR upon intravenous administration of peptide at 30 mg/kg; nt: Not tested in spontaneously hypertensive rats.

## 4. Antihypertensive Peptides from Fish Sources

ACE inhibitory peptides from fish sources were first identified in sardine meat over twenty years ago [90]. Since then ACE inhibitory peptides have been found in various fish species, including shellfish, tuna, bonito, salmon and sardine [49,72,73,74,75,91].

There have been several reports of crude fish protein hydrolysates containing ACE inhibitory peptides. Following the hydrolysis of purified catfish protein with the commercial enzyme Protamex^®^ ACE inhibitory peptides were released, with the majority being found in the soluble protein fraction [92]. A crude enzyme extract from the viscera of sardine was used to hydrolyze the protein contained within the head and viscera of the fish species know as sardinelle. The resulting hydrolysate contained a high concentration of peptides displaying low hydrophobicity with molecular masses between 200 and 600 Da [93]. Pacific Hake fish protein subjected to simulated gastrointestinal digestion was reported to contain ACE inhibitory activity. Most inhibitory peptides were reported to be short chained, polar and containing few hydrophobic amino acids in their sequence [94].

### Antihypertensive Peptides from Fish By-Products

A major issue for food producers is discarded by-products of food processing. Adding value to waste streams is very appealing to food producers, as the by-products are usually incorporated into low economic value products such as animal feed. The frame protein of Alaskan Pollock which is normally discarded as an industrial by-product was mined for ACE inhibitory peptides [95]. Frame protein was first hydrolyzed with pepsin and then separated into five fractions depending on molecular weight, the most active ACE inhibitory peptides were found in the fraction <1 kDa. From this fraction, a novel peptide was isolated with an amino acid sequence FGASTRGA and an IC_50_ value of 14.7 µM.

The normally discarded yellowfin sole frame protein was identified as another source of ACE inhibitory peptides [96]. Using α-chymotrypsin, a peptide with a molecular mass of 1.3 kDa was isolated with the corresponding amino acid sequence MIFPGAGGPEL. It was demonstrated that this peptide reduced the SBP of SHR over 9 h when administrated at 10 mg per kilogram of animal body weight. The SBP of SHR was reduced by 22 mmHg at 3 h, a reduction which was comparable to the group treated with Captopril. Reduction in SBP remained after 9 h, suggesting that MIFPGAGGPEL is an effective antihypertensive agent *in vivo*. Frame protein hydrolysate derived from tuna frame protein was investigated for novel ACE inhibitory peptides [51]. Six proteases were used, with pepsin hydrolysates displaying the highest ACE inhibitory activity. Consecutive purification steps resulted in the isolation of an ACE inhibitory peptide of 21 amino acids in length. The peptide with the amino acid sequence GDLGKTTTVSNWSPPKYKDTP had an IC_50_ value of 11.28 µM and actively decreased the SBP of SHR for up to 9 h after oral administration, with a maximum decrease of 21 mmHg after 6 h.

Following oral administration of a thermolysin hydrolysate from chum salmon muscle, the SBP of SHR was significantly reduced when compared to that of the controls. The greatest reduction in SBP occurred after 4 h suggesting that short chain peptides were responsible for the blood pressure lowering effect, since reduction in blood pressure occurred soon after administration. Indeed, a reduction in SBP 6 to 8 h after administration is associated with peptides of greater chain length, because larger peptides are converted from substrate to true ACE inhibitors *in vivo* [74]. The SBP of all test groups returned to initial levels after 24 h, demonstrating that hydrolysates of chum salmon are temporary hypotensors. The amino acid sequences of the six di-peptides identified from the thermolysin digest of chum salmon were WA, VW, WM, MW, IW, and LW. The IC_50_ values of these six peptides were determined as 277.3, 2.5, 98.6, 9.8, 4.7 and 17.4 µM, respectively. Interestingly, it was found that a change in the amino acid sequence of some dipeptides resulted in a change in the inhibitory mechanism as well as a change in the reported IC_50_ value [97]. For example, the peptide MW has an IC_50_ value of 9.8 µM and is a non-competitive inhibitor of ACE. When the amino acid sequence was changed to WM, the IC_50_ value increased to 98.6 µM and mode of inhibition changed to competitive, indicating that the amino acid composition of peptides as well as position of amino acids in the peptide sequence contributes to the ACE inhibitory activity of peptides. 

The pepsin hydrolysate of Bigeye tuna dark muscle was reported to contain the ACE inhibitory peptide WPEAAELMMEVDP, with a molecular mass of 1581 Da and an IC_50_ value of 21.6 µM [49]. Employing Lineweaver-Burk plots, the authors determined that the peptide forms enzyme-substrate-inhibitor and enzyme-inhibitor complexes to lower the efficiency of ACE *in vitro*. Administration of this peptide to SHR resulted in a maximum decrease in SBP between 3 and 6 h. The SBP of test animals remained lower than control animals (~15 mmHg) after 10 h proving that WPEAAELMMEVDP is an effective hypotensor *in vivo*.

Following the hydrolysis of shark meat with the protease SM98011 from *Bacillus* sp. SM98011, four ACE inhibitory peptides were identified [98]. Three of the peptides were reported as being novel ACE inhibitory peptides, the amino acid sequences of these peptides were EY, FE and CF, with IC_50_ values of 1.98, 2.68 and 1.45 µM, respectively.

Fujita *et al.* [72] developed a thermolysin hydrolysate from “Katsuo-bushi”, a traditional Japanese food processed from dried bonito. This hydrolysate was administered to thirty hypertensive and borderline hypertensive human subjects in a small scale clinical trial. The hydrolysate contained the previously reported ACE inhibitory peptide LKPNM [75]. Following eight weeks of administration of the dried bonito hydrolysate, the SBP of subjects was reduced by 12.55 ± 1.5 mmHg. This clearly demonstrates that the thermolysin hydrolsate of dried bonito is sufficiently well absorbed in humans to effectively reduce the SBP of hypertensive and borderline hypertensive subjects *in vivo*. The hydrolysate has been approved as Foods for Specified Health Use (FOSHU) by the Ministry of Health and Welfare in Japan [72]. A summary of ACE inhibitory fish derived peptides is given in Table 2.

**Table 2:** ACE inhibitory peptides derived from meat of fish. Adapted with permission from American Chemical Society [3], Copyright 2005.

Source	Amino acid sequence	Parent protein	Enzyme	IC_50_ (µM)	SHR ^a^	Ref.
bonito	IKPLNY	muscle	thermolysin	43	nt	[75]
	IVGRPRHQG	actin	thermolysin	2.4	nt	[75]
	IWHHT	actin	thermolysin	5.8	nt	[75]
	ALPHA	actin	thermolysin	10	nt	[75]
	FQP	actin	thermolysin	12	nt	[75]
	LKPNM	muscle	thermolysin	2.4	∆ 23.0 mmHg ^b^	[72,75]
	IY	actin	thermolysin	2.31	∆ 19.0 mmHg ^b^	[72,75]
	DYGLYP	muscle	thermolysin	62	nt	[75]
	LKP	muscle	thermolysin	0.32	∆ 18.0 mmHg ^b^	[72]
	IWHHT	actin	thermolysin	3.5	∆ 26.0 mmHg ^b^	[72]
	IKP	muscle	thermolysin	6.9	∆ 20.0 mmHg ^b^	[72]
	IVGRPR	actin	thermolysin	300	∆ 25.0 mmHg ^b^	[89]
salmon	WA	muscle	thermolysin	277.3	nt	[74]
	VW	muscle	thermolysin	2.5	nt	[74]
	WM	muscle	thermolysin	96.6	nt	[74]
	MW	muscle	thermolysin	9.9	nt	[74]
	IW	muscle	thermolysin	4.7	nt	[74]
	LW	muscle	thermolysin	17.4	nt	[74]
sardine	MF	muscle	alcalase	44.7	nt	[73]
	RY	muscle	alcalase	51	nt	[73]
	MY	muscle	alcalase	193	nt	[73]
	LY	muscle	alcalase	38.5	nt	[73]
	YL	muscle	alcalase	82	nt	[73]
	IY	muscle	alcalase	10.5	nt	[73]
	VF	muscle	alcalase	43.7	nt	[73]
	GRP	muscle	alcalase	20	nt	[73]
	RFP	muscle	alcalase	330	nt	[73]
	AKK	muscle	alcalase	3.13	nt	[73]
	RVY	muscle	alcalase	205.6	nt	[73]
	GWAP	muscle	alcalase	3.86	nt	[73]
	KY	muscle	alcalase	1.63	nt	[73]
	VY	muscle	alcalase	10	∆ 7.0 mmHg	[99,100]
tuna	PTHIKWGD	muscle	acid	nd	nt	[101]
	GDLGKTTTVSNWSPPKYKDTP	frame protein	pepsin	11.28	∆ 21.0 mmHg	[51]
	WPEAAELMMEVDP	dark muscle	pepsin	21.6	∆ 18.0 mmHg	[49]
alaska pollack	GPL	skin	alcalase + pronase + collagenase	2.65	nt	[102]
	GPM	skin	alcalase + pronase + collagenase	17.13	nt	[102]
	FGASTRGA	frame protein	pepsin	14.7	nt	[95]
yellowfin sole	MIFPGAGGPEL	frame protein	α chymotrypsin	28.7	∆ 22.0 mmHg	[96]
shark	EY	muscle	protease SM98011	1.98	nt	[98]
	FE	muscle	protease SM98012	2.68	nt	[98]
	CF	muscle	protease SM98013	1.45	nt	[98]

^a^ The maximum decrease in systolic blood pressure in SHR upon oral administration of peptide at 10 mg/kg;

^b^ The maximum decrease in systolic blood pressure in SHR upon oral administration of peptide at 60 mg/kg; nt: Not tested in SHR; nd: IC_50_ of peptide was not determined.

## 5. Antioxidant Peptides

Antioxidants are known to be beneficial to human health as they may protect the body against molecules known as reactive oxygen species (ROS), which can attack membrane lipids, protein and DNA. This in turn can be a causative factor in many diseases such as cardiovascular disease, diabetes, cancer and Alzheimer’s disease. Lipid oxidation can cause deterioration of food quality and a reduction in the shelf-life of a food product, while the consumption of foods containing lipid oxidation products has been linked to various diseases, including cancers, diabetes and cardiovascular disease [103,104,105,106].

The use of natural antioxidants in foodstuffs is appealing because of the potential health risk associated with synthetic antioxidants *in vivo* [107,108,109,110]. Antioxidant peptides have been found in numerous foodstuffs including milk [111], wheat [16], potato [22] and fungi [112]. In the past number of years, a great deal of research has focused on antioxidant peptides sourced from fish, while the research on antioxidant peptides from the hydrolysates of domesticated animal muscle is limited. There are a number of methods available to measure antioxidant potential of various food components based on the ability of potential antioxidants to scavenge ROS, free radicals or prevent oxidation in model systems, some of which are discussed below.

### 5.1. Antioxidant Peptides from Fish Sources

The hydrolysates of mackerel were found to contain peptides exhibiting antioxidant activity *in vitro* [113]. Peptides released by the hydrolysis of mackerel with Protease N inhibited the autoxidation of linoleic acid, quenched the free radical α,α-diphenyl-β-picrylhydrazyl (DPPH) and reduced Fe^3+^ to Fe^2+^. The strongest antioxidant fraction of mackerel protein hydrolysate contained small peptides and free amino acids and had a molecular weight of 1400 Da.

Alaska Pollack frame protein (APF) was found to contain the peptide LPHSGY, released after hydrolysis of APF by a crude enzyme extract contained within mackerel intestine. This peptide was responsible for the quenching of 35% of available hydroxyl radicals at a peptide concentration of 53.6 µM. The authors proposed that a correlation existed between the observed antioxidant activity and the molecular weight of the peptides, with greater antioxidant activity associated with peptides of low molecular weight (<1 kDa) [114].

The antioxidant potential of Tilapia (common name for cichlid fish) protein hydrolysates was also demonstrated [115]. Hydrolysates generated with the enzymes Cyrotin-F, Protease A, Protease N, Flavourzyme and Neutrase showed significant ability to scavenge ROS and reduce ferric ions. The ability to effectively scavenge ROS was associated with the low molecular weight peptides present in the hydrolysates. Low molecular weight peptides were also responsible for the observed antioxidant activity in the hydrolysates of Silver carp following hydrolysis by Alcalase and Flavourzyme [116]. In previous studies, it was noted that increasing the degree of hydrolysis produced greater numbers of low molecular weight peptides, which was associated with an increase in the radical scavenging ability of salmon muscle hydrolysate [97]. This phenomenon, however, was not observed in the hydrolysates of catfish protein isolate hydrolyzed with Protamex [92], where the ability of the hydrolysates to scavenge DPPH radicals or reduce Fe^3+^ decreased with increasing degree of hydrolysis. An increase in the number of low molecular weight peptides did; however, result in an increase in the time required for lipid peroxidation products to accumulate in a model muscle washed system. This suggests that antioxidant ability of peptides *in vitro* depends on peptide size, amino acid composition of the peptide and presence of free amino acids within the hydrolysate.

The peptides from Alcalase- and Flavourzyme-digested Yellow Stripe Trevally were shown to prevent oxidative damage to DNA using the Fenton reaction. The possible reason for this was that hydrolysates chelated Fe^2+^ thus preventing it from reacting with H_2_O_2_ and forming hydroxyl radicals [117].

Seven antioxidant peptides were identified in the hydrolysates of proteins recovered from the waste stream of sardinelle processing [118]. The amino acid sequences of the seven peptides were LARL, GGE, LHY, GAH, GAWA, PHYL and GALAAH. All peptides were <600 Da and were deemed to be novel antioxidant peptides. It was reported that the peptide LHY displayed the highest DPPH radical scavenging activity, with an ability to scavenge 63% of available DPPH radical present at a peptide concentration of 150 µg/mL.

Antioxidant peptides were generated from the hydrolysis of tuna dark muscle by-product by the commercial enzymes orientase (OR) and protease XXІІІ (PR) [119]. Antioxidant activity was determined by measuring the DPPH radical scavenging capacity and the ability of hydrolysates to inhibit or delay lipid peroxidation of linoleic acid. From the OR enzyme digest, a purified antioxidant peptide with the amino acid sequence LPTSEAAKY was identified as having the capacity to scavenge 79.6% of available DPPH radicals, while also inhibiting lipid peroxidation of linoleic acid for 7.13 days at a peptide concentration of 100 µg/mL. Following the hydrolysis of tuna dark muscle by-product with the enzyme PR, the peptide with amino acid sequence PMNYMVT was demonstrated to scavenge 85.2% of available DPPH radicals, while also inhibiting oxidation of linoleic acid for 7.89 days at a peptide concentration of 100 µg/mL. Furthermore, when compared to the controls (butyl hydroxyanisol (BHA) and α-tocopherol employed in determination of lipid peroxidation inhibition, and L-ascorbic acid in determination of DPPH radical scavenging ability), both peptides displayed similar if not higher activity. Therefore, it is clear that the peptides generated during the hydrolysis of tuna dark muscle by-product are effective *in vitro* antioxidants with potential for incorporation into foodstuffs as natural antioxidants. 

Hoki skin gelatin was hydrolyzed by the digestive enzymes pepsin, trypsin and α-chymotrypsin with the tryptic hydrolysates displaying the highest levels of antioxidant activity [120]. A single purified peptide, with the amino acid sequence HGPLGPL was identified as being responsible for the observed antioxidant activity of the tryptic hydrolysate. When the peptide HGPLGPL was added to a linoleic acid peroxidation system, inhibition of lipid peroxidation was significantly greater than that of the control α-tocopherol and as effective as the synthetic antioxidant butylated hydroxytoluene (BHT). Furthermore, the peptide HGPLGPL increased the expression of antioxidative enzymes in human hepatoma cells *in vitro*.

### 5.2. Antioxidant Peptides from Meat Sources

Peptides derived from porcine myofibrillar proteins using the proteases Papain and Actinase E represent the first report of antioxidant peptides from the myofibrillar proteins of edible meat [121]. Following digestion, these crude hydrolysates inhibited peroxidation of linoleic acid, DPPH scavenging and metal chelating activities. Purification of the peptides responsible for the antioxidant activity in the Papain hydrolysate resulted in the identification of five peptides: DSGVT (actin), IEAEGE (unknown), DAQEKLE (tropomyosin), EELDNALN (tropomyosin) and VPSIDDQEELM (myosin heavy chain). 

A cocktail of enzymes including pepsin, papain and proteases from the bovine pancreas and bacterial proteases from *Streptomyces* and *Bacillus polymyxa*, were used to hydrolyze porcine collagen [122]. This resulted in the release of the antioxidant peptide QGAR, which was identified as residues 72–75 and 180–183 of the α1 chain of collagen. It was proposed that the QG amino acid residues may be a contributing factor to radical scavenging activity.

Plasma proteins represent another valuable but under-utilized protein source. Indeed, following hydrolysis with Alcalase, porcine plasma hydrolysates (PPH) were found to be effective inhibitors of lipid oxidation, as well as effective DPPH scavengers, metal chelating and reducing agents [123]. Increasing the degree of hydrolysis increased antioxidant activity and the most active fractions were <3 kDa. The peptide with MW 441 Da and amino acid sequence HNGN was identified as the active antioxidant peptide.

Chicken essence, a traditional product consumed in China was shown to possess various antioxidant activities including the inhibition of linoleic acid autoxidation, DPPH radical scavenging activity, reducing power and the ability to chelate metal ions [124]. From the extract of chicken essence, two peptides were identified that displayed inhibition of the autoxidation of linoleic acid in a model system. The first peptide with the amino acid sequence HVTEE had an induction period of 2.49 days while the second peptide with the amino acid sequence PVPVEGV had an induction period of 6.50 days.

A hydrolysate displaying strong antioxidant activity was produced following the digestion of venison with papain where two peptides responsible for the activity were identified, *i.e.*, (APVPH І) MQIFVKTLTG and (APVPH ІІ) DLSDGEQGVL [7]. The peptide APVPH І displayed the greater free radical scavenging activity of the two peptides with IC_50_ values of 4.47, 7.82, 22.02 and 8.62 µM per mL for hydroxyl, DPPH, superoxide and peroxyl radicals, respectively, lower than the control IC_50_ value of vitamin C.

## 6. Antimicrobial and Antiproliferative Peptides

Antimicrobial peptides (AMPs) have been identified in a range of foods to date, with peptides released from milk proteins being the most plentiful source of AMPs. Indeed, AMPs have been found in a host of different milk proteins including the caseins [125,126] and lactoferrin [127,128]. While AMPs from the protein of muscle foods are less well documented, there has been one report of AMPs from a bovine meat source [10]. In this study, the antimicrobial and human cancer cell cytotoxic effects of four previously identified ACE inhibitory peptides, were evaluated. The four peptides GFHI, DFHING, FHG and GLSDGEWQ were assayed for antimicrobial activity against six pathogenic bacteria, three Gram-positive (*Bacillus cereus*, *Listeria monocytogenes* and *Staphylococcus aureus*) and three Gram-negative (*Salmonella typhimurium*, *Escherichia coli* and *Pseudomonas aeruginosa*). The peptide GLSDGEWQ inhibited the growth of *S. typhimurium*, *B. cereus*, *E. coli* and *L. monocytogenes*. This was the only peptide that inhibited the growth of both Gram-positive and Gram-negative pathogens. GFHI and FHG inhibited the growth of the pathogen *P. aeruginosa*.

A cysteine rich antimicrobial peptide was produced from the digestion of oyster muscle using a combination of Alcalase and bromelin [26]. The peptide referred to as *CgPep33* inhibited the growth of the pathogenic bacteria *E. coli*, *P. aeruginosa*, *B. subtilis* and *S. aureus* in addition to inhibiting the growth of the fungi *Botrytis cinerea* and *Penicillium expansum*. Although the sequence of the *CgPep33* peptide was not determined, the principal amino acids present in the active fraction were C, L, E, D, F, Y, I and G residues. Jang *et al.* [10] investigated the cytotoxic effect of four AMPs from a bovine meat source, using the cell lines breast adenocarcinoma (MCF-7), stomach adenocarcinoma (AGS) and lung carcinoma (A549) cells. The peptide GFHI possessed the strongest cytotoxic effect on MCF-7 cells and also decreased the cell viability of AGS cells, while the peptide GLSDGEWQ strongly inhibited the proliferation of AGS cells.

Peptides isolated from anchovy sauce were capable of inducing apoptosis in a human lymphoma cell line (U937) [129]. The peptide of interest was characterized as being hydrophobic and having a molecular weight of 440.9 Da [130]. Protein hydrolysates from different fish species were investigated for their antiproliferative activity on two human breast cancer cell lines (MCF-7/6 and MDA-MB-231) [131]. Hydrolysates of blue whiting, cod, plaice and salmon resulted in the significant inhibition of growth in both MCF-7/6 and MDA-MB-231 cell lines.

Recently, the hydrolysate of tuna dark muscle by-product was examined for potential antiproliferative activity by exposure to the human breast cancer cell line MCF-7 [132]. Peptide fractions within the molecular weight range of 400 and 1400 Da exhibited the strongest antiproliferative activity. In these fractions two antiproliferative peptides were identified, *i.e.*, LPHVLTPEAGAT from papain hydrolysate and PTAEGVYMVT from Protease XXIII. The IC_50_ value of each peptide was determined to be 8.1 and 8.8 µM, respectively. This demonstrates once again the potential of meat products and meat by-products as valuable sources of bioactive peptides for incorporation into functional foods. 

## 7. Conclusion

The increase in food related diseases, such as cardiovascular disease, diabetes, cancer and obesity has led consumers to demand food products that not only offer nutritional value but also functional and health benefits. Meat and fish derived peptides have been shown to exhibit antihypertensive effects *in vivo*, along with antioxidant capabilities and other bioactivities such as antimicrobial and antiproliferative activities *in vitro*. In spite of this; however, very few food products containing meat or fish-derived bioactive peptides are available commercially, yet these bioactives harbor huge potential. For example, Katsuobushi Oligopeptide LKPNM, which is found in blood pressure-lowering capsules, is derived from dried bonito and converted to the active form by digestive enzymes following ingestion [133]. The commercial chicken-meat extract known as “Brand’s Essence of Chicken” (BEC; Cerebos Pacific Ltd., Singapore) also claims to contain antihypertensive components [134]. These two examples show the potential of fish and meat bioactive peptides for use as functional ingredients in foods. However, the ability of bioactive peptides to exert a physiological effect *in vivo* is dependent on the bioavailability of the peptide. This factor is dependent on the resistance of the peptide to hydrolysis by peptidases of both the intestinal tract and serum, and its ability to be absorbed across the intestinal epithelium [61]. Therefore, when identifying bioactive peptides for the development of meat and fish-based nutraceutical products, this fact should be taken into account.
